# Cardiometabolic Risk Clusters and Their Reproductive Correlates: A Latent Class Analysis of Indian Women

**DOI:** 10.5334/gh.1408

**Published:** 2025-03-11

**Authors:** Wilhemina Quarpong, Suchitra Chandrasekaran, K. M. Venkat Narayan, Usha Ramakrishnan, Nikhil Tandon, Shivani A. Patel

**Affiliations:** 1 Nutrition and Health Sciences, Laney Graduate School, Emory University, Atlanta, Georgia, USA; 2 Department of Gynecology and Obstetrics, Emory University, Atlanta, Georgia, USA; 3 Emory Global Diabetes Research Center of Woodruff Health Sciences Center and Emory University, Atlanta, Georgia, USA; 4 Hubert Department of Global Health, Rollins School of Public Health, Emory University, Atlanta, Georgia, USA; 5 Department of Endocrinology and Metabolism, All India Institute of Medical Sciences, New Delhi, India

**Keywords:** Cardiometabolic health, metabolic dysfunction, cardiovascular disease, reproductive history, Indian women

## Abstract

**Background:**

Cardiometabolic conditions are rising among women in low- and middle-income countries and appearing at younger ages. The role of female reproductive characteristics in cardiometabolic risk is not well understood.

**Methods:**

We analyzed seven reproductive characteristics and seven cardiometabolic indicators obtained from 644,191 non-pregnant women aged 15–49 years in the 2019–2021 India National Family and Health Survey (NFHS-5). We conducted a latent class analysis of cardiometabolic indicators (systolic and diastolic blood pressure, random blood glucose, body mass index, waist circumference, and use of anti-hyperglycemic and antihypertensive pharmacotherapy) to identify risk clusters. Multinomial logistic regression models accounting for age and sociodemographic characteristics assessed associations between reproductive characteristics (age at menarche, age at first birth, natural or surgical menopause, parity, time since last birth, experience of pregnancy loss, current contraceptive use) and cluster membership.

**Results:**

Women had a median age of 29.4 (IQR: 21.5–38.4) years, were mostly married (71%), and resided in rural areas (68%). Five cardiometabolic clusters emerged: normal (36%), high-normal (46%), isolated-overweight (12%), hypertension-overweight (6%), and glucose dysregulation-overweight (1%). Early menarche (<13 years), early age at first birth (<20 years), and natural or surgical menopause were positively associated with two or more high-risk clusters (ORs: 1.13–1.62). Higher parity was associated with higher relative odds of isolated-overweight (ORs: 1.31–1.39), while longer time since last birth (≥ 8 years) was associated with hypertension-overweight (OR: 1.25 95% CI: 1.18–1.31) and glucose dysregulation-overweight (OR: 1.21, 95% CI: 1.07–1.37). Pregnancy loss increased the odds of all high-risk clusters (ORs: 1.21–1.42), while contraceptive use decreased the odds (ORs: 0.88–0.93).

**Conclusions:**

Five cardiometabolic risk clusters were identified in Indian women, with cluster membership linked to reproductive characteristics. The timing of fertility milestones and reproductive history appear relevant for early risk stratification among women in early to middle adulthood.

**Key Messages:**

- Indian women aged 15–49 years exhibited 5 distinct patterns of cardiometabolic risk clustering: normal, high-normal, isolated-overweight, hypertension-overweight, and glucose dysregulation-overweight clusters.
- Early age at menarche (<13 years), early age at first birth (<20 years), natural or surgical menopause, higher parity, longer time since last birth, and pregnancy losses were associated with at least one of the high-risk cardiometabolic clusters.
- Reproductive history and the timing of reproductive milestones may improve early disease risk stratification in Indian women.

## Introduction

Cardiometabolic conditions—most prominently high blood glucose, high body mass index, dyslipidemia, and high blood pressure—are rising globally, posing significant threats to health and longevity (1,2,3). These conditions are not only preventable but also medically treatable. Understanding how these interrelated conditions manifest and cluster within populations can provide insights into disease etiology, informing prevention and management strategies for a broad spectrum of health issues (4,5,6). Of particular concern is the disproportionate burden of multiple cardiometabolic conditions among women, who remain underrepresented in epidemiologic research (7,8,9). Insights into the clustering patterns of these conditions among women are crucial for developing tailored health strategies.

The distinct reproductive biology of females may increase their susceptibility to co-occurring cardiometabolic conditions. Emerging evidence (10,11,12) suggests that hormonal changes related to sexual maturation, pregnancy and breastfeeding, and menopause may each contribute to the development of cardiometabolic dysregulation in women. However, existing research (13,14,15,16,17) on the association of reproductive characteristics with women’s cardiometabolic health has produced conflicting findings regarding the magnitude and direction of some of these associations. Moreover, most studies have been conducted in high-income countries among women of European descent, who are generally at lower risk of cardiometabolic risk compared with other ethnic groups (18).

Investigating the relationship between reproductive characteristics and clustering of cardiometabolic conditions is needed in India, where women exhibit a concerning rising burden of cardiometabolic dysfunction, even at normal body weight (19), along with higher rates of reproductive morbidities compared to other populations (11). With projections (20,21) indicating a continued rise in these issues, elucidating the role of fertility milestones and reproductive history in the development of cardiometabolic dysregulation presents a unique opportunity for early risk screening and disease prevention in this population (11).

Using nationally representative data on 644,191 women aged 15 to 49 years from the India National Family and Health Survey (NFHS-5), we aimed to identify clusters of cardiometabolic risk factors and assess associations between reproductive characteristics and cardiometabolic risk clusters.

## Methods

### Study Population

We analyzed cross-sectional survey data on women aged 15–49 years participating in the fifth round of the India National Family and Health Survey (NFHS-5) (22). The India NFHS is a large-scale multi-round survey that employs complex sampling procedures to recruit participants, ensuring representativeness at the national, regional, and residence levels. The survey targets women of reproductive age and collects essential data on their health and family welfare via interviews and physical examinations. NFHS-5 was conducted between 2019 and 2021 and recruited 724,115 participants through a stratified two-stage cluster design, with women sampled from households embedded within clusters of rural and urban communities (22). Data were available upon request from the Demographic and Health Surveys Program: www.dhsprogram.com.

We restricted our analysis to non-pregnant women aged 15–49 years who were free of heart disease and had at least one of the cardiometabolic measures needed for the study (systolic and diastolic blood pressure, random non-fasting blood glucose, body mass index, and waist circumference).

### Study measures

#### Cardiometabolic measures

Seven cardiometabolic indicators were available in the NFHS-5 survey: systolic blood pressure, diastolic blood pressure, random blood glucose, body mass index, waist circumference, and use of anti-hyperglycemic and/or antihypertensive pharmacotherapy.

Systolic and diastolic blood pressure were measured at three time points with an interval of five minutes between readings using the Omron Blood Pressure Monitor (22). We used the average of the second and third blood pressure measurements.

Blood glucose measures were taken at the participant’s home using an Accu-Chek Performa glucometer with a finger-stick blood specimen (22). A fasted state was not required for the glucose test (22); therefore, for uniformity, we restricted our analysis to participants with random blood glucose who comprised the larger proportion (94%) of the sample.

Body mass index (BMI) was calculated as an individual’s weight in kilograms divided by the square of their height in meters. Height and weight were measured using the Seca 213 stadiometer and the Seca 874 digital scale, respectively (22). Waist circumference was measured using Gulick tape measures (22).

The use of anti-hyperglycemic and antihypertensive pharmacotherapy was self-reported by participants.

#### Reproductive characteristics

Seven self-reported reproductive characteristics with putative associations with cardiometabolic health were available in the NFHS-5 survey. We considered three measures of reproductive milestones: age at menarche (dichotomized at the median as <13 years or ≥13 years), age at first birth (dichotomized at the median as <20 years or ≥20 years), and menopausal status (defined as natural or surgical menopause). Four measures of reproductive history were available: parity (categorized as nulliparous, one-to-two, and three or more births), time since last birth (dichotomized at the median as <8 years or ≥8 years), experience of pregnancy loss (miscarriage, abortion, or stillbirth), and current contraceptive use (categorized as none or modern and/or traditional).

#### Sociodemographic covariates

We considered the following sociodemographic covariates in analyses: age, education (categorized as lower than secondary, secondary, and higher than secondary education), household wealth index (categorized into five quintiles), marital status (categorized as never married, currently married and together, and ever married but currently apart, i.e., either widowed, divorced, separated, or deserted), urbanicity (categorized into urban and rural), and religion (categorized as Hindu, Muslim, Christian, and other religions).

#### Health behaviors

Our analyses accounted for three health behaviors that are established risk factors for cardiometabolic health, including current alcohol consumption, current use of any tobacco products, and daily consumption of fruits and dark green, leafy vegetables.

### Statistical Analyses

#### Descriptive analyses

The distribution of sociodemographic characteristics of the study population was estimated after accounting for the survey design. Weighted prevalence with 95% confidence intervals and medians with interquartile ranges (IQR) were reported for categorical and continuous variables, respectively.

#### Latent class analysis to identify cardiometabolic clusters

Latent Class Analysis (LCA) was conducted to identify discrete patterns, which we refer to as clusters, in the co-occurrence of the seven cardiometabolic measures. The model was selected to balance the tradeoff between parsimony and model fit. Model fit was assessed using the following relative fit indicators – Akaike Information Criterion (AIC), Bayes Information Criterion (BIC), Sample Size-Adjusted Bayesian Information Criterion (SABIC), and entropy. Models with lower AIC, BIC, and SABIC values and larger entropy indicated a better fit. The appropriate number of classes was evaluated using the Lo-Mendell-Rubin Adjusted Likelihood Ratio Test (adjusted-LMR). A p-value less than 0.05 for the adjusted LMR indicated that the model under consideration (k class model) was a better fit compared to a k-1 class model. We also compared latent class proportions across models to ensure that the selected model had a reasonable number of participants within each class. Average latent class probabilities for the most likely latent class membership were also compared across models to ensure that members within each class of the identified model had high probabilities of belonging to their allocated class. Finally, we considered the interpretability of competing models to ensure that identified classes were coherent cardiometabolic groupings.

We explored a series of latent class models, ranging from one to six classes, and selected a five-class model to be the best fit for the data based on lower AIC, BIC, and SABIC values relative to lower-class models (Supplementary Table 2). Although a six-class model had lower AIC, BIC, and SABIC values than the five-class model, the adjusted LMR likelihood ratio test indicated that the additional class did not significantly improve the model. In addition, the five-class model had better class separation than the six-class model (entropy of 0.783 compared to 0.759). These five classes were treated as cardiometabolic risk clusters in the remainder of the analyses.

Identified clusters (latent classes) of risk factors from the selected model were labeled based on the variation in the observed statistical means of cardiometabolic measures within the cluster. We described clusters with an average systolic blood pressure value ≥140 mmHg and/or average diastolic blood pressure value ≥90 mmHg as having hypertension (i.e., stage 2 hypertension and above) (23). Clusters with an average random blood glucose ≥200 mg/dl were described as having a sign of glucose dysregulation (24). Clusters that had an average BMI ≥25 kg/m^2^ were described as clusters with overweight/obesity (25). High visceral fat was based on an average waist circumference >85 cm.

We also calculated predicted marginal means of the continuous cardiometabolic measures and average predicted probabilities of pharmacotherapy use for each cluster, adjusting for sociodemographic characteristics (age, education, wealth, marital status, urbanicity, and religion) and health behaviors (current alcohol consumption, current use of any tobacco products, and daily consumption of fruits and dark green, leafy vegetables).

While data on one or more cardiometabolic measures were missing for 4% of women, the pattern of missingness was determined to be at random, conditional on observed covariates. We therefore applied Full Information Maximum Likelihood (FIML) estimation during LCA to utilize data from all participants who had at least one cardiometabolic measure.

#### Reproductive characteristics and cardiometabolic clusters

We described the sociodemographic and reproductive characteristics composition of each cardiometabolic cluster. Rao-Scott chi-squared tests and survey-adjusted models accounting for the cluster design were used to assess the significance of differences in characteristics between cardiometabolic clusters.

We employed multinomial logistic regression models to estimate the unadjusted and adjusted association between each reproductive factor of interest and cluster membership. For these models, we combined the lowest risk clusters (clusters that had the lowest mean level of the continuous indicators) and used them as the reference category, reporting the odds of being in each of the “higher” risk clusters (three clusters), relative to being in the lowest risk clusters (two combined clusters).

LCA was conducted using Mplus Version 8.10. All other statistical analyses were conducted using the SAS programming software, version 9.4.

## Results

### Sociodemographic and reproductive characteristics

**Figure 1:**
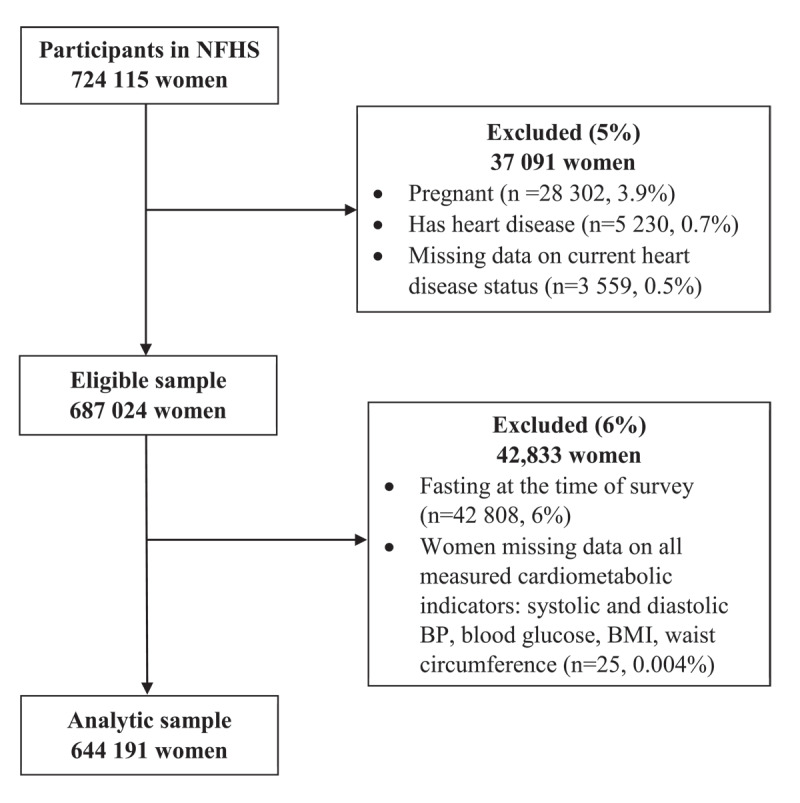
Flowchart of women (age 15–49 years) participating in the India National and Family Health Survey, NFHS-5 2019–2021. Data on 644,191 women were analyzed after the exclusion of pregnant women, women with heart disease at the time of survey, and women missing data on all measured cardiometabolic indicators: systolic and diastolic blood pressure, blood glucose, body mass index, and waist circumference.

Of 724,115 women aged 15–49 years who participated in the survey, 28,302 (3.9%) were pregnant at the survey, and 8,789 (1.2%) had heart disease or were missing information on current heart disease status and were thus ineligible for this study. Of 687,024 women who were eligible for this analysis (Figure 1), 42,808 (6%) women who were fasting at the time of the survey and 25 (0.004%) women who were missing information on all the measured cardiometabolic indicators were excluded from analysis, resulting in an analytic sample of 644,191 women. Characteristics of women excluded from the analysis are shown in Supplementary Table 1.

Women in our analytic sample (N = 644,191) had a median (IQR) age of 29.4 (21.5–38.4) years (Table 1). The majority (68.1%) resided in rural areas, had at least a secondary school education (65.5%), and were currently married and living with their partners (71.3%).

**Table 1:** Sociodemographic and reproductive characteristics of Indian women, NFHS-5 2019–2021 (N = 644,191).


CHARACTERISTIC	UNWEIGHTED SAMPLE SIZE	WEIGHTED PREVALENCE (%) OR MEDIAN	95% CI OR IQR

**Sociodemographic**			

Age (years), median	644,191	29.4	21.5, 38.4

Education, %			

None or lower than secondary	226,042	34.5	34.3, 34.8

Secondary	329,116	50.3	50.1, 50.5

Higher than secondary	89,033	15.2	15.0, 15.4

Marital status, %			

Never married	166,699	24.4	24.2, 24.5

Currently married and together	449,724	71.3	71.1, 71.4

Ever married, but currently apart^a^	27,768	4.4	4.3, 4.4

Place of residence, %			

Urban	157,959	31.9	31.5, 32.2

Rural	486,232	68.1	67.8, 68.5

**Reproductive**			

Age at menarche (15–24 years subsample, n = 210,743), median	208,848	12.9	12.2, 13.7

Age at menarche (15–24 years subsample, n = 210,743), %			

<13 years	40,753	17.3	16.9, 17.6

≥13 years	168,095	82.7	82.4, 83.1

Age at first birth (years), median	443,255	19.6	17.5, 22.1

Age at first birth, %			

<20 years	181,058	43	42.8, 43.3

≥20 years	262,197	57	56.7, 57.2

Menstrual status, %			

Currently menstruating	549,309	84.9	84.7, 85.0

Lactational amenorrhea or birth within 6 months	19,325	2.8	2.8, 2.9

Absence of period due to unspecified reasons	31,809	5.2	5.1, 5.3

Natural/surgical menopause	41,853	6.8	6.7, 6.9

Never menstruated	1,895	0.3	0.3, 0.3

Parity, median	644,191	1.2	0.0, 2.2

Parity, %			

Nulliparous	200,936	30.0	29.9, 30.2

1–2 births	251,808	41.2	41.0, 41.4

≥3 births	191,447	28.7	28.6, 28.9

Time since last birth, %			

0–7 years	222,497	49.1	48.8, 49.3

≥8 years	220,758	50.9	50.7, 51.2

Experience of pregnancy loss,^b^ %	71,969	12.2	12.1, 12.3

Current use of traditional or modern contraceptives^c^	326,885	52.5	52.3, 52.7


^a^Either widowed, divorced, separated, or deserted. ^b^Pregnancy that resulted in a miscarriage, abortion, or stillbirth, i.e., did not result in a live birth. ^c^Traditional contraceptive methods included the folkloric method, withdrawal, and periodic abstinence. Modern contraceptive methods included pills, IUDs, injections, diaphragms, condoms, sterilization, emergency contraception, standard days method, lactational amenorrhea, and foam and jelly.

The median (IQR) age at menarche (15–24 years subsample, n = 210,743) was 12.9 (12.2–13.7) years. Among women who had ever given birth, the median (IQR) age at first birth was 19.6 (17.5–22.1) years. The majority (84.9%, 95% CI: 84.7–85.0%) of women were currently menstruating; 2.8% (95% CI: 2.8–2.9%) were postpartum amenorrhoeic or had had a birth within six months; 5.2% (95% CI: 5.1–5.3) had not menstruated for at least 12 months for unspecified reasons; 6.8% (95% CI: 6.7–6.9%) had natural or surgical menopause; and 0.3% (95% CI: 0.3–0.3%) had never menstruated.

The median (IQR) parity was 1.2 (0–2.2) births; 30.0% (95% CI: 29.9–30.2%) of women reported no births, and 41.2% (95% CI: 41.0–41.4%) reported one or two births. About 49% (95% CI: 48.8–49.3%) of the women had a birth within seven years before the survey, and 50.9% (95% CI: 50.7–51.2%) had a birth eight years or more prior to the survey. Twelve percent of the women had lost at least one pregnancy through miscarriage, abortion, or stillbirth. Current traditional and/or modern contraceptive use in the population was 52.5% (95% CI: 52.3–52.7%).

### Characteristics of cardiometabolic risk clusters

**Figure 2:**
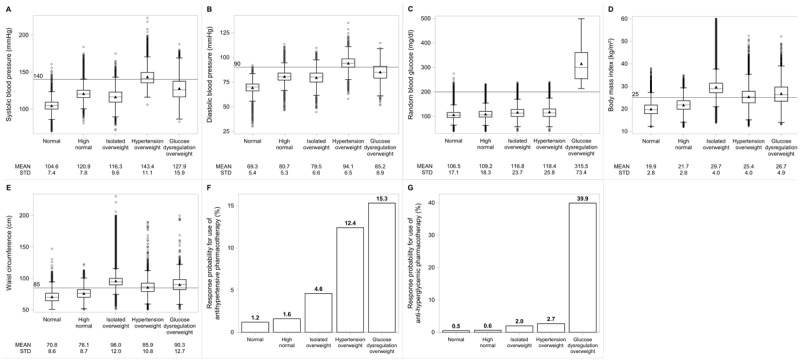
Cardiometabolic characteristics of Indian women by latent cluster, NFHS-5 2019–2021 (N = 644,191). Distributions of cardiometabolic measures [systolic blood pressure **(A)**, diastolic blood pressure **(B)**, random blood glucose **(C)**, body mass index **(D)**, and waist circumference **(E)**] and response probabilities for antihypertensive **(F)** and anti-hyperglycemic **(G)** pharmacotherapy use across cardiometabolic clusters.

Identified clusters were categorized as normal (cluster one), high-normal (cluster two), isolated-overweight (cluster three), hypertension-overweight (cluster four), and glucose dysregulation-overweight (cluster five) clusters. Figure 2 shows the observed means and response probabilities of the cardiometabolic measures across clusters, while Table 2 shows the predicted marginal means and average response probabilities, accounting for sociodemographic factors and health behaviors.

**Table 2:** Characteristics of cardiometabolic clusters of Indian women, NFHS-5 2019–2021 (N = 644,191).


CARDIOMETABOLIC CHARACTERISTIC	CLUSTER					

	CLUSTER 1: NORMAL	CLUSTER 2: HIGH-NORMAL	CLUSTER 3: ISOLATED-OVERWEIGHT	CLUSTER 4: HYPERTENSION-OVERWEIGHT	CLUSTER 5: GLUCOSE DYSREGULATION-OVERWEIGHT	P-VALUE^b^

Prevalence of class, n (%)	229,892 (35.7%)	294,325 (45.7%)	74,036 (11.5%)	40,344 (6.3%)	5,594 (0.9%)	

Predicted marginal mean^a^ (95% CI)						

Systolic blood pressure (mmHg)	104.5 (104.3, 104.7)	120.3 (120.1, 120.5)	115.5 (115.3, 115.7)	142.3 (142.1, 142.5)	126.4 (125.8, 127.0)	<.0001

Diastolic blood pressure (mmHg)	69.8 (69.7, 69.9)	80.9 (80.8, 81.1)	79.5 (79.4, 79.7)	93.9 (93.7, 94.0)	84.8 (84.4, 85.1)	<.0001

Random blood glucose (mg/dl)	107.9 (107.4, 108.3)	108.6 (108.1, 109.0)	115.4 (114.9, 115.9)	115.5 (114.9, 116.0)	309.3 (306.7, 311.9)	<.0001

Body mass index (kg/m^2^)	20.2 (20.1, 20.2)	21.6 (21.5, 21.6)	29.0 (29.0, 29.1)	24.9 (24.8, 25.0)	26.0 (25.8, 26.1)	<.0001

Waist circumference (cm)	72.1 (71.9, 72.3)	76.1 (75.9, 76.3)	94.2 (93.9, 94.4)	84.7 (84.4, 85.0)	88.3 (87.8, 88.8)	<.0001

Average predicted response probability^a^, (95% CI)						

Use of antihypertensive pharmacotherapy	1.4 (1.2, 1.6)	1.4 (1.2, 1.5)	3.5 (3.1, 4.0)	9.0 (8.1, 10.1)	9.5 (8.3, 10.9)	<.0001

Use of anti-hyperglycemic pharmacotherapy	0.5 (0.4, 0.7)	0.5 (0.4, 0.6)	1.6 (1.3, 2.0)	2.1 (1.7, 2.6)	28.0 (23.6, 32.8)	<.0001


^a^Models are adjusted for age, education, wealth, marital status, urbanicity, religion, and health behaviors (tobacco and alcohol use and fruit and vegetable consumption). ^b^T-test p-values for predicted marginal means and Rao-Scott adjusted p-values for predicted probabilities.

The normal cluster (35.7%) had mean cardiometabolic measures within normal ranges for all the continuous indicators. The high-normal cluster (45.7%) also had cardiometabolic measures within normal ranges, yet higher on average than the normal cluster. The isolated-overweight cluster (11.5%), on average, had normal systolic and diastolic blood pressure values, with observed mean BMI (29.7 kg/m^2^) and waist circumference (96.0 cm) in the overweight range. In contrast, the hypertension-overweight cluster (6.3%) had mean BMI (25.4 kg/m^2^) and waist circumference (85.9 cm) in the overweight range but mean systolic (143.4 mmHg) and diastolic (94.1 mmHg) blood pressure in the hypertension range. The highest mean random blood glucose was observed in the glucose dysregulation-overweight cluster. Women in this cluster (0.9%) had a mean random blood glucose (315.5 mg/dl) in the diabetes range. They also had the second-highest mean values for BMI (26.7 kg/m^2^) and waist circumference (90.3 cm). The lowest response probabilities for antihypertensive and anti-hyperglycemic pharmacotherapy use were observed in the normal cluster (1.2% and 0.5%, respectively). Pharmacotherapy use was highest in the glucose dysregulation-overweight cluster, with 40% and 15% reporting the use of anti-hyperglycemic and antihypertensive pharmacotherapy, respectively.

### Associations between reproductive factors and latent class membership

The results of multinomial logistic regression analysis of reproductive factors and cardiometabolic risk clusters are shown in Table 3. In fully adjusted models controlling for age, sociodemographic factors, other reproductive measures, and health behaviors, each reproductive characteristic was associated with being in at least one high-risk cluster.

**Table 3:** Multinomial logistic regression analysis of reproductive factors and cardiometabolic risk clusters among Indian women, NFHS-5 2019–2021 (N = 644,191).


CHARACTERISTIC	UNADJUSTED PREVALENCE ODDS RATIO (95% CI) [REFERENCE= NORMAL AND HIGH-NORMAL CLUSTERS]			^a^ADJUSTED PREVALENCE ODDS RATIO (95% CI) [REFERENCE= NORMAL AND HIGH-NORMAL CLUSTERS]		
	
	CLUSTER 3: ISOLATED-OVER-WEIGHT	CLUSTER 4: HYPER-TENSION-OVER-WEIGHT	CLUSTER 5: GLUCOSE DYSRE-GULATION-OVERW-EIGHT	CLUSTER 3: ISOLATED-OVER-WEIGHT	CLUSTER 4: HYPER-TENSION-OVER-WEIGHT	CLUSTER 5: GLUCOSE DYSRE-GULATION-OVERW-EIGHT

**Reproductive milestones**						

Age at menarche (ref =≥ 13 years)^b^						

<13 years	**1.55 (1.43, 1.67)**	1.18 (0.99, 1.39)	1.51 (0.92, 2.49)	**1.62 (1.49, 1.75)**	**1.21 (1.02, 1.43)**	1.54 (0.94, 2.54)

Age at first birth (ref = ≥ 20 years)						

<20 years	**0.85 (0.83, 0.88)**	**1.10 (1.06, 1.13)**	**1.24 (1.15, 1.34)**	**1.15 (1.12, 1.18)**	**1.23 (1.19, 1.28)**	**1.53 (1.41, 1.66)**

Menstrual status (ref = menstruating)						

Absence of period due to unspecified reasons	**1.46 (1.39, 1.53)**	**2.48 (2.35, 2.61)**	**3.01 (2.67, 3.40)**	**1.09 (1.03, 1.15)**	**1.10 (1.04, 1.16)**	**1.32 (1.16, 1.50)**

Natural/surgical menopause	**1.93 (1.85, 2.00)**	**4.23 (4.01, 4.40)**	**5.15 (4.70, 5.64)**	1.04 (1.00, 1.09)	**1.13 (1.08, 1.18)**	**1.35 (1.21, 1.49)**

**Reproductive history**						

Parity (ref = nulliparous)						

1–2 births	**3.96 (3.82, 4.11)**	**5.14 (4.85, 5.44)**	**5.76 (5.04, 6.58)**	**1.39 (1.31, 1.48)**	1.01 (0.92, 1.10)	0.96 (0.80, 1.16)

≥ 3 births	**3.31 (3.12, 3.44)**	**7.33 (6.93, 7.74)**	**7.25 (6.36, 8.27)**	**1.31 (1.23, 1.40)**	0.93 (0.85, 1.02)	0.87 (0.72, 1.05)

Time since last birth (ref = <8 years)						

≥8 years	**1.76 (1.72, 1.80)**	**4.60 (4.44, 4.77)**	**5.63 (5.10, 6.20)**	0.99 (0.95, 1.03)	**1.25 (1.18, 1.31)**	**1.21 (1.07, 1.37)**

Experience of pregnancy loss (ref = no)^c^						

Yes	**1.82 (1.76, 1.87)**	**1.66 (1.59, 1.73)**	**2.00 (1.82, 2.20)**	**1.30 (1.26, 1.34)**	**1.21 (1.16, 1.26)**	**1.42 (1.29, 1.57)**

Current contraceptive use (ref = none)						

Traditional or modern^d^	**1.99 (1.94, 2.04)**	**2.23 (2.16, 2.30)**	**2.16 (2.00, 2.33)**	**0.92 (0.89, 0.95)**	**0.93 (0.90, 0.97)**	**0.88 (0.81, 0.97)**


Statistically significant results are in bold. ^a^Models are adjusted for other reproductive factors (parity, current breastfeeding status, experience of pregnancy loss, and menopausal/hysterectomy status), sociodemographic factors (age, education, wealth index, marital status, urbanicity, and religion), and health behaviors (alcohol and tobacco use and fruit and vegetable consumption). ^b^Analysis restricted to women aged 15–24 years, n = 210,743. ^c^Pregnancy that resulted in a miscarriage, abortion, or stillbirth, i.e., did not result in a live birth. ^d^Traditional contraceptive methods included the folkloric method, withdrawal, and periodic abstinence. Modern contraceptive methods included pills, IUDs, injections, diaphragms, condoms, sterilization, emergency contraception, standard days method, lactational amenorrhea, and foam and jelly.

With respect to reproductive milestones, early age at menarche (<13 years) was positively associated with being in the isolated-overweight cluster (OR = 1.62, 95% CI: 1.49–1.75) and the hypertension-overweight cluster (OR = 1.21, 95% CI 1.02–1.43) but not the glucose dysregulation-overweight cluster (OR = 1.54, 95% CI: 0.94–2.54) in adjusted models. Age at first birth <20 years was associated with 15–53% higher odds of belonging to a high-risk cluster [OR = 1.15 (95% CI: 1.12–1.18) for the isolated-overweight cluster and 1.53 (95% CI: 1.41–1.66) for the glucose dysregulation-overweight cluster]. Finally, natural or surgical menopause was positively associated with being in the hypertension-overweight (OR = 1.13, 95% CI: 1.08–1.18) and glucose dysregulation-overweight clusters (OR = 1.35, 95% CI: 1.21–1.49).

Regarding reproductive history, parity was positively associated with the isolated-overweight cluster [ORs of 1.39 (95% CI: 1.31–1.48) for one-to-two births and 1.31 (95% CI: 1.23–1.40) for ≥3 births] but not with the other clusters. A last birth ≥8 years before the survey was positively associated with being in the hypertension-overweight (OR = 1.25, 95% CI: 1.18–1.31) and glucose dysregulation-overweight (OR = 1.21, 95% CI: 1.07–1.37) clusters. Experiencing pregnancy losses (miscarriage, stillbirth, or abortion) was associated with 21–42% higher odds of belonging to a high-risk cluster. In contrast, the use of any form of contraception was negatively associated with all the high-risk clusters. The odds ratios (95% CI) for the associations between current contraceptive use and membership in the isolated-overweight, hypertension-overweight, and glucose dysregulation-overweight clusters were 0.92 (0.89, 0.95), 0.93 (0.90–0.97), and 0.88 (0.81–0.97) respectively.

## Discussion

In this large, nationally representative study of Indian women aged 15 to 49 years, we investigated cardiometabolic clusters and their reproductive correlates. First, we identified five distinct clusters of cardiometabolic health. Nearly one-fifth of the population was classified within high-risk clusters, with 12% classified as women with isolated-overweight, 6% as women with hypertension-overweight, and 1% as women with glucose dysregulation-overweight. Second, we found that three reproductive milestones—early age at menarche, early age at first birth, and menopause—and three reproductive history measures—parity, longer time since last birth, and pregnancy losses—were linked to a higher likelihood of belonging to one or more of the identified high-risk cardiometabolic clusters. Collectively, the findings suggest the importance of the reproductive lifecourse in the early development of cardiometabolic conditions.

Rather than studying cardiometabolic indicators in isolation, our study evaluated the distribution of coexisting cardiometabolic risks. A key finding was the consistent presence of overweight across all high-risk clusters, including hypertension and diabetes. This aligns with literature describing overweight as a precursor to other cardiometabolic syndrome components (26). Being overweight induces inflammatory cycles, disrupting endocrine and metabolic functions, leading to systemic insulin resistance and vascular dysfunction (26).

Among the reproductive milestones investigated, menarche and menopause have been most studied in relation to cardiometabolic health. Early menarche, often indicative of early sexual maturation, is linked to childhood obesity, which may persist into adulthood (12,27,28), raising the risk of further cardiometabolic disorders. Menopause results in reduced estrogen levels, which heightens the risk of cardiometabolic dysfunction, especially during the menopausal transition, which is characterized by systemic inflammation, weight gain, elevated blood pressure, and glucose intolerance (29). Similar to other studies (27,30), we found a positive association between early menarche and isolated-overweight and hypertension-overweight clusters. The relationship between age at menarche and glucose dysregulation in adulthood is not consistent, and we did not find an association in our study. Menopause, on the other hand, was not associated with isolated-overweight but was associated with both the hypertension-overweight and glucose dysregulation-overweight clusters. This supports previous theories that obesity mediates the relationship between menarche and cardiometabolic health, while the link between menopause and cardiometabolic health appears to be obesity independent.

Menarche and menopause define the reproductive lifespan and relate to early childbearing and total pregnancies. Pregnancy triggers significant physiological changes that, while essential for a healthy pregnancy and normally reversible, may negatively impact future cardiometabolic outcomes (12). Early menarche correlated with early childbearing in our study and has been reported in the literature (31). Women experiencing early childbirth may face increased cardiometabolic risks due to multiple pregnancies over their reproductive lifespan (12). As demonstrated in our study, women with multiple pregnancies had higher odds of being overweight.

History of pregnancy loss was associated with the isolated-overweight, hypertension-overweight, and glucose dysregulation-overweight clusters, possibly reflecting residual effects from pregnancy-related physiological changes or later effects of pregnancy complications. Alternatively, the association might indicate reverse causation, where women with pre-existing cardiometabolic disorders are more susceptible to pregnancy loss. Few studies have examined this issue (32). While the mechanism for this specific association is unclear, studies report shared underlying mechanisms between pregnancy loss and cardiometabolic measures such as endothelial dysfunction, impaired glucose tolerance, and chronic systemic inflammation (32,33).

While any form of contraception appeared to reduce the likelihood of being in a high-risk group, this association may have been influenced by the predominance of non-hormonal methods (>80%) in the study population, as these are generally not associated with increases in weight, blood pressure, or blood glucose (34,35,36). Our findings contrast with research from high-income countries, where hormonal contraceptive methods are more common, and their impact on cardiometabolic risk has been well-documented (36,37). Our study population, which predominantly uses non-hormonal contraception, differs significantly from those in high-income settings, highlighting the need for more research to understand this association in diverse populations.

In addition to the physiological mechanisms explaining the connections between reproductive events and increased cardiometabolic risks, these events have also been linked to a heightened risk of psychological stressors and lifestyle and socioeconomic changes, which may contribute to the accumulation of cardiometabolic risks over time (38,39).

Our study has several strengths, notably a large, nationally representative sample of young to middle-aged women, enhancing generalizability to this population. The data provide insights into the clustering of risk factors for cardiometabolic disease in this under-researched demographic, utilizing objective measures. The use of LCA to identify subgroups of women with distinct patterns of cardiometabolic clusters is another strength, given that this method has several advantages over traditional clustering methods (40). Additionally, the inclusion of a wide range of reproductive factors offers a comprehensive overview of their associations with cardiometabolic health.

Limitations of this study include its cross-sectional design, which precludes investigating longitudinal associations of reproductive factors and future cardiometabolic health. The absence of dyslipidemia measures in NFHS-5 limited our ability to estimate comprehensive cardiometabolic risk and may underestimate the burden of cardiometabolic clustering. Self-reported reproductive histories may have introduced recall bias.

## Conclusion

Our findings demonstrate the presence of cardiometabolic risk clustering in a relatively younger population of women, implying that adverse measures emerge relatively early in life, even in middle-income country settings. The differing clustering patterns observed can support the implementation of screening, prevention, management, and treatment strategies that target multiple risk factors or specific risk factor combinations.

Our epidemiologic analysis indicated that the timing of reproductive milestones and reproductive history played a role in the cardiometabolic health status of women by middle age. Notably, we observed positive associations between specific reproductive factors and these clusters, including early age at menarche, early age at first birth, menopause, parity, time since last birth, and pregnancy loss. This underscores the importance of considering reproductive history in cardiometabolic disease risk assessment. Further studies investigating the temporality of these associations, underlying mechanisms, and associations between the identified risk factor clusters and future cardiometabolic disease are needed.

## Data Accessibility Statement

The data that support the findings of this study are available upon request from the Demographic and Health Surveys Program: www.dhsprogram.com.

## Additional Files

The additional files for this article can be found as follows:

10.5334/gh.1408.s1 Supplementary Table 1. Differences in sociodemographic and lifestyle characteristics between analytic and excluded sample.

10.5334/gh.1408.s2 Supplementary Table 2. Fit statistics and class proportions for LCA models on cardiometabolic risk factors among women (N = 644,191) participating in the 2019–2021 India National Family and Health Survey.
