# Supplementary material for: Cardiometabolic Risk Clusters and Their Reproductive Correlates: A Latent Class Analysis of Indian Women
Source: Glob Heart. 2025 Mar 11;20(1):25. doi: 10.5334/gh.1408 (PMC11908431; doi:10.5334/gh.1408)
Supplement: Supplementary Table 1. — Differences in sociodemographic and lifestyle characteristics between analytic and excluded sample. [file gh-20-1-1408-s1.pdf]

Cardiometabolic risk clusters and their reproductive correlates among Indian women

Supplementary table 1. Differences in sociodemographic and lifestyle characteristics between analytic and excluded sample

	Weighted % or mean (95% CI)		
**Characteristic**	Analytic sample (n=644,191)	Excluded sample (n=79,924)	**P-value**
Sociodemographic			
Age (years)	30.6 (30.6, 30.7)	28.9 (28.8, 29.0)	<.0001
Education			<.0001
None or lower than secondary	34.5 (34.3, 34.8)	31.4 (30.8, 31.9)	
Secondary	50.3 (50.1, 50.5)	49.6 (49.0, 50.1)	
Higher than secondary	15.2 (15.0, 15.4)	19.1 (18.5, 19.7)	
Wealth quintile			<.0001
Lowest	18.4 (18.2, 18.7)	19.1 (18.6, 19.5)	
Second	20.2 (20.0, 20.4)	18.3 (17.9, 18.8)	
Middle	20.8 (20.6, 21.1)	18.2 (17.7, 18.7)	
Fourth	20.9 (20.7, 21.2)	19.9 (19.3, 20.4)	
Highest	19.6 (19.3, 19.9)	24.5 (23.7, 25.4)	
Marital status			<.0001
Never married	24.4 (24.2, 24.5)	19.1 (18.6, 19.5)	
Currently married and together	71.3 (71.1, 71.4)	77.6 (77.1, 78)	
Ever married, but currently apart[a]	4.4 (4.3, 4.4)	3.4 (3.2, 3.6)	
Religion			<.0001
Hindu	82.0 (81.6, 82.4)	76.8 (76.0, 77.6)	
Muslim	13.0 (12.6, 13.4)	17.2 (16.4, 17.9)	
Christian	2.3 (2.2, 2.4)	2.5 (2.3, 2.7)	
Other[b]	2.7 (2.6, 2.8)	3.5 (3.1, 3.8)	
Place of residence			<.0001
Urban	31.9 (31.5, 32.2)	37.0 (36.2, 37.9)	
Rural	68.1 (67.8, 68.5)	63.0 (62.1, 63.8)	
Health behaviors			
Tobacco use	4.1 (4.0, 4.2)	3.7 (3.5, 3.8)	0.1200
Alcohol use	0.8 (0.7, 0.8)	0.7 (0.6, 0.8)	0.0047
Less than daily fruit and vegetable consumption	92.1 (92.0, 92.3)	88.4 (88.0, 88.8)	<.0001

[a]Either widowed, divorced, separated, or deserted.
[b]Other category includes Sikh, Buddhist/Neo-Buddhist, Jain, Jewish, Parsi/Zoroastrian, and no religion.
