# Supplementary material for: Cardiometabolic Risk Clusters and Their Reproductive Correlates: A Latent Class Analysis of Indian Women
Source: Glob Heart. 2025 Mar 11;20(1):25. doi: 10.5334/gh.1408 (PMC11908431; doi:10.5334/gh.1408)
Supplement: Supplementary Table 2. — Fit statistics and class proportions for LCA models on cardiometabolic risk factors among women (N = 644,191) participating in the 2019–2021 India National Family and Health Survey. [file gh-20-1-1408-s2.pdf]

Supplementary table 2. Fit statistics and class proportions for LCA models on cardiometabolic risk factors among women (N=644,191) participating in the 2019-2021 India National Family and Health Survey

	Model					
**Fit statistic**	**1-class**	**2-class**	**3-class**	**4-class**	**5-class**	**6-class**
AIC	24548834.77	24099085.78	23755705.99	23605203.52	23464938.21	23394746.85
BIC	24548971.28	24099313.30	23756024.51	23605613.05	23465438.74	23395338.39
SABIC	24548933.14	24099249.74	23755935.53	23605498.64	23465298.90	23395173.14
Adjusted LMR	-	0	0	0	0	0.3333
Entropy	-	0.777	0.850	0.785	0.783	0.759
Class membership						
Class 1	644,191 (100%)	488,620 (75.9%)	477,357 (74.10%)	391,064 (60.1%)	294,325 (45.7%)	232,511 (36.1%)
Class 2		155,571 (24.2%)	160,979 (24.99%)	170,473 (26.5%)	229,892 (35.7%)	212,065 (32.9%)
Class 3			5,855 (0.91%)	76,844 (11.9%)	74,036 (11.5%)	128,610 (20.0%)
Class 4				5,810 (0.9%)	40,344 (6.3%)	43,162 (6.7%)
Class 5					5,594 (0.9%)	22,385 (3.8%)
Class 6						5,458 (0.8%)

Latent class indicators: systolic blood pressure, diastolic blood pressure, blood glucose, body mass index, waist circumference, use of glucose pharmacotherapy, use of antihypertensive pharmacotherapy. Abbreviations: AIC - Akaike Information Criterion; BIC - Bayesian Information Criterion; SABIC – Sample Size-Adjusted Bayesian Information Criterion; LMR - Lo-Mendell-Rubin Adjusted Likelihood Ratio Test; BLRT - Bootstrapped Likelihood Ratio Test.
